# Primary somatosensory neuron-derived Zn^2+^ orchestrates muscle regeneration via Hippo signaling pathway

**DOI:** 10.1038/s41421-026-00910-8

**Published:** 2026-07-01

**Authors:** Fang Tong, Jingjing Zhou, Ziyue Chen, Xiaocui Tang, Xicheng Gu, Wenjie Wang, Huan Yang, Ping Hu, Zuoyun Wang, Qingjian Han

**Affiliations:** 1 Shanghai Pudong Hospital, Fudan University Pudong Medical Center, State Key Laboratory of Brain Function and Disorders and MOE Frontiers Center for Brain Science, Institutes of Brain Science; Department of Anatomy and Histoembryology, School of Basic Medical Sciences, Fudan University, Shanghai, China; 2 Precision Research Center for Refractory Diseases, Institute for Clinical Research, Shanghai General Hospital, Shanghai Jiao Tong University School of Medicine, Shanghai, China; 3 Department of Gastroenterology, Huadong Hospital Affiliated to Fudan University, Shanghai, China; 4 Key Laboratory of Biological Targeting Diagnosis, Therapy and Rehabilitation of Guangdong Higher Education Institutes, the Fifth Affiliated Hospital of Guangzhou Medical University, Guangzhou, Guangdong, China; 5 Guangzhou Laboratory, Guangzhou International Bio Island, Guangzhou, Guangdong China; 6 Bayinguoleng Mongolia Autonomous Prefecture Hygiene School, Korla, Xinjiang Uygur Autonomous Region China

**Keywords:** Muscle stem cells, Adult stem cells

Dear Editor,

Skeletal muscle is a striated muscle tissue that is anatomically attached to bones via tendons and is densely innervated by nerve fibers from both motor neurons (for voluntary contraction) and somatosensory neurons (for proprioceptive feedback and somatosensation)^1^. The extraordinary regenerative capacity of skeletal muscle ensures its recovery from injury, preserves mobility, and maintains metabolic health, making it essential for longevity and physical performance. Elucidating the mechanism underlying muscle regeneration could reveal new therapeutic strategies for muscle-degenerative diseases and age-related functional decline. Clinical evidence indicates that disruption of neural innervation, as observed in spinal cord injury, precipitates rapid muscle atrophy, whereas interventions that stimulate neural activity — such as functional electrical stimulation, stretching, massage, resistance training, and vibration — effectively mitigate muscle loss and promote regeneration. Collectively, these findings highlight the critical role of the nervous system in maintaining muscle homeostasis and regeneration. However, the mechanisms by which the nervous system, particularly somatosensory neurons, orchestrate muscle regeneration remain largely unknown.

In the process of regeneration, muscle stem cells (MuSCs, also called muscle satellite cells) play an indispensable role. Under physiological conditions, MuSCs maintain in an undifferentiated state and are marked as Pax7^+^ cells^2,3^. Upon injury, MuSCs become activated from quiescence, upregulating myogenic regulators such as MyoD, MyoG, and MyHC, while downregulating the Pax7 expression^4^. The newly formed myofibers either fuse with each other or with the original myofibers to form regenerated skeletal muscle. Several studies have shown that primary somatosensory nerves can play a regulatory role in stem cells activity, such as in haematopoietic stem cells and epidermal stem cells^5,6^. Here, we aim to explore the role of primary somatosensory nerve in muscle regeneration and to elucidate the specific molecular mechanisms involved.

To explore the role of somatosensory neurons in muscle regeneration, we first examined the innervation of the tibialis anterior (TA) muscle under normal physiological conditions. Immunofluorescence staining revealed that the TA muscle is extensively innervated by low-threshold mechanoreceptors (LTMRs), marked by Neurofilament 200 (NF200) and tyrosine hydroxylase (TH). In contrast, nociceptors labeled by calcitonin gene-related peptide (CGRP) and purinergic receptor P2X ligand-gated ion channel 3 (P2X_3_), were relatively sparse (Supplementary Fig. S1a). Notably, dorsal root ganglia (DRG) sections were included as positive controls, confirming the specificity and staining efficiency of each neuronal marker. Furthermore, we crossed flox-tdTomato-flox reporter mice with various neuron type-specific Cre lines to label different subtypes of sensory neurons innervating TA, including Aβ-LTMRs (Vglut1-Cre; Ai14), C-LTMRs (TH-CreERT; Ai27), pan-primary somatosensory neurons (Advillin-Cre; Ai27), and nociceptors (SNS-Cre; Ai3). Consistently, we observed the presence of LTMR innervation in the TA (Supplementary Fig. S1b). Cardiotoxin (CTX) injections were administered to induce muscle injury in TA. Immunofluorescence staining was performed on the cryosections from TA at 0, 5, 7, and 21 days post-CTX treatment. The number of Pax7^+^ MuSCs increased significantly, accompanied by the rise of NF200^+^ nerve fiber number in TA (Supplementary Fig. S2). To comprehensively understand the distribution of NF200^+^ nerve fibers and MuSCs in the TA muscle, and to assess their spatial relationship and potential interactions, we performed clearing using clear lipid-exchanged acrylamide-hybridized rigid imaging/immunostaining-compatible tissue hYdrogel (CLARITY) technique and stained the cleared tissues with anti-NF200 (red) and anti-Pax7 (green). MuSCs seemed to be located in the close proximity of the nerve fibers (Fig. 1a, b; Supplementary Fig. S3a–c). The distribution of NF200^+^ nerve fibers, MuSCs, and their spatial distances were further analyzed using 3D remodeling (Fig. 1b; Supplementary Fig. S3d). CTX-treated muscles exhibited a significant increase in NF200^+^ nerve fibers and Pax7⁺ cells compared with vehicle controls. Moreover, the distance between MuSCs and NF200^+^ nerve fibers was markedly reduced while the branch level of dendrite increased following CTX treatment (Fig. 1b; Supplementary Fig. S3c–f). These findings suggest that NF200^+^ nerve fibers may regulate MuSCs and contribute to muscle regeneration. The TA muscle is innervated by the sciatic nerve, which contains both afferent fibers transmitting somatosensory signals to the central nervous system and efferent fibers conveying motor commands to the muscle. Somatosensory neurons have their cell bodies located in the DRG, which lies just outside the spinal cord near the dorsal horn. The peripheral processes of these DRG neurons extend distally toward peripheral tissues, while their central processes project into the dorsal horn of the spinal cord. In contrast, motor neurons have their cell bodies located in the ventral horn of the spinal cord and send efferent axons outward to innervate skeletal muscle. Distally, the sensory afferent fibers originating from the DRG merge with motor efferent fibers from the ventral horn to form the mixed sciatic nerve. To selectively eliminate somatosensory input of TA muscle, we performed rhizotomy by transecting the distal afferent branch of the L4–5 DRG before it merges with the motor efferent fibers to form the sciatic nerve using Pax7-EGFP mice (Fig. 1c; Supplementary Fig. S4a). Fluoro-Gold retrograde tracing performed in the ipsilateral plantar region confirmed that the afferent branch was completely disconnected, while the efferent branch remained intact (Fig. 1d; Supplementary Fig. S4a, b). Four weeks later, CTX was injected into both TA muscles at both the denervation side and the intact side to induce muscle damage. The subsequent immunofluorescence staining analysis performed at 5 days post-injury revealed a smaller regenerative area in the TA muscle on the rhizotomized side (Fig. 1e, f; Supplementary Fig. S4c, d). To test whether Zn²⁺ mediates this effect, we intrathecally administered the Zn^2+^ chelator ZX1 2 days before CTX-induced injury to disrupt local Zn^2+^ availability within the sensory pathway. TA muscles were collected at 5 days post-injury for analysis. ZX1 treatment similarly impaired TA muscle regeneration (Supplementary. Fig. 4e, f). Additionally, we performed intramuscular injection of ZX1 into the TA muscle to chelate local Zn^2+^, including Zn^2+^ released from sensory nerve terminals. TA muscles were harvested at 5 days post-injury and analyzed by Laminin immunofluorescence staining. We observed that local administration of ZX1 significantly suppressed muscle regeneration (Supplementary Fig. S4g, h). These findings are consistent with a model in which sensory neuron-derived Zn^2+^ contributes to efficient muscle regeneration.

**Fig. 1:**
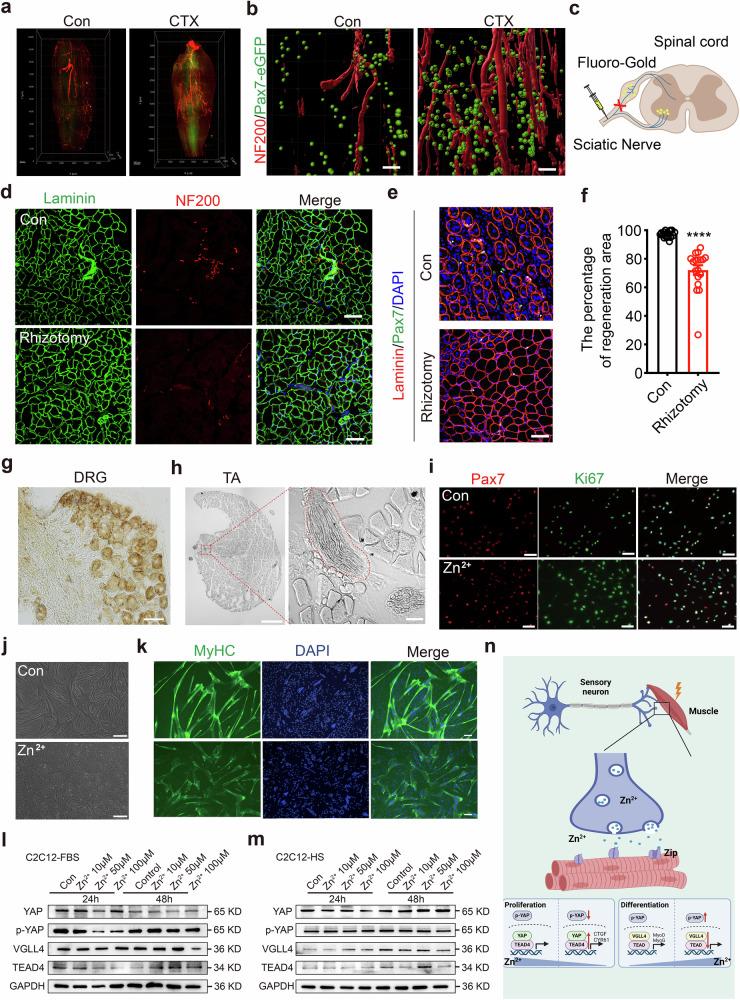
Sensory neurons promote muscle regeneration through Zn^2+^ by regulating Hippo pathway. **a** 3D images of TA on both sides of Pax7-EGFP mice after tissue clearing. Scale bars, 1000 μm. **b** 3D modeling of sensory nerve and MuSCs. Green: Pax7^+^ cells; Red: NF200^+^ nerve fibers. Scale bars, 200 μm. **c** Schematic diagram of rhizotomy and fluorogold injection. **d** Representative immunofluorescence (IF) image of sensory nerve in TA after rhizotomy. Scale bars, 100 μm. **e** Cross-sectional comparison of TA muscle of rhizotomy model after muscle injury. Scale bars, 100 μm. **f** Quantification of TA muscle regeneration area following ablation of sensory nerve fibers (****P < 0.0001, Unpaired *t*-test). **g** Zn^2+^ staining of DRG. Scale bar, 50 μm. **h** Zn^2+^ staining of TA. The right figure is a partial enlargement of the dotted line in the left figure. Scale bar, 500 μm in left panel and 25 μm in right panel. **i** The representative IF images of Pax7 and Ki67 in human MuSCs under the action of Zn^2+^ (concentration: 100 μM). Scale bar, 50 μm. **j** Phase contrast images of human MuSCs after 48 h differentiation under Zn^2+^ treatment (100 μM). Scale bar, 200 μm. **k** The representative IF images of MyHC in human MuSCs under Zn^2+^ treatment (100 μM). Scale bar, 50 μm. **l** Changes in Hippo pathway protein expression during proliferation under Zn^2+^ treatment. **m** The protein changes of Hippo pathway during differentiation. **n**. Schematic diagram of sensory neurons regulating muscle regeneration through Zn^2+^.

Next, we explored the mechanism for how the somatosensory nerves regulate muscle regeneration. Previous studies demonstrated that NF200^+^ somatosensory neurons contain abundant Zn^2+^ within vesicles at their axon terminals, which is released upon stimulation^7^. Zn^2+^ is an essential trace element that plays a multifaceted andindispensable role in the regulation of growth and regeneration. Clinical studies have shown that restoring zinc levels can improve wound healing, muscle recovery after injury, and even neural tissue repair. Thus, we investigated whether Zn^2+^ mediated the interaction between nerve fibers and MuSCs. Using Zns^AMG^ staining, we found that Zn^2+^ is enriched in a subset of neurons in L4–5 DRG that give rise to the sciatic nerve and innervate the TA muscle (Fig. 1g, h). To date, a total of 14 Zn^2+^ transporters, which belong to Zrt- and Irt-related protein (ZIP) family, have been identified. These proteins facilitate the transport of Zn^2+^ from the extracellular space into the cytosol^8^. We examined the expression of ZIPs in MuSCs by reanalyzing a published single-cell RNA sequencing (scRNA-seq) dataset. ZIP1 (*Slc39a1*), ZIP6 (*Slc39a6*), ZIP7 (*Slc39a7*), ZIP9 (*Slc39a9*), ZIP10 (*Slc39a10*), ZIP13 (*Slc39a13*), and ZIP14 (*Slc39a14*) are all expressed in MuSCs (Supplementary Fig. S5a–c). Consistent with the expression in MuSCs, the ZIP family members were also abundantly expressed in the mouse myoblast cell line C2C12 (Supplementary Fig. S5d, e). These findings suggest that Zn^2+^ released from somatosensory nerve terminals may regulate muscle regeneration by modulating MuSC proliferation and differentiation of MuSCs via ZIPs expressed on their membranes.

To further understand the mechanism for how Zn^2+^ released from somatosensory neuron terminals regulates muscle regeneration, we examined its effect on human MuSC and mouse myoblast C2C12 cells. Zn^2+^ treatment promoted the proliferation of MuSCs (Fig. 1i; Supplementary Fig. S6), while inhibited the differentiation, as evidenced by the downregulation of the differentiation marker MyHC (Fig. 1j–k; Supplementary Fig. S7). Hippo pathway involves a variety of biological processes, including organ growth, embryogenesis, and tissue regeneration via regulating cell proliferation, differentiation, and apoptosis^9^. Our previous studies revealed that the Hippo signaling pathway (YAP and VGLL4) played a dual role in MuSCs proliferation and differentiation. Based on this, we speculate whether Zn^2+^ regulates the proliferation and differentiation of MuSCs via the Hippo signaling pathway. We next investigated whether Hippo signaling was activated by Zn^2+^. The results showed that Zn^2+^ led to the decrease of p-YAP and VGLL4, resulting in the promotion of cell proliferation by YAP during the proliferation stages of C2C12 cells (Fig. 1l); while in the differentiation stage, p-YAP upregulated and Zn^2+^ treatment reduced VGLL4 protein level, which caused a delay in differentiation (Fig. 1m). To determine whether the effects of Zn^2+^ on C2C12 cell proliferation depend on Hippo signaling pathway, we treated C2C12 cells with vehicle, Zn^2+^, or Zn^2+^ in combination with the YAP inhibitor verteporfin to inhibit Hippo pathway activity. The results showed that verteporfin treatment effectively blocked Zn^2+^-induced activation of the Hippo/YAP signaling pathway (Supplementary Fig. S8a). Co-treatment with Zn^2+^ and verteporfin abolished the Zn^2+^-mediated alterations in Hippo signaling. The upregulation of p-YAP and VGLL4 suggested an attenuated pro-proliferative activity of the nuclear YAP (Supplementary Fig. S8a). In line with our previous work showing that the Hippo pathway regulated skeletal muscle repair and regeneration following injury via modulating MyoD and MyoG expression and function^10^, these results established that Zn^2+^-mediated promotion of C2C12 cell proliferation is mediated through the canonical Hippo-YAP signaling axis. Consistent with the changes in the Hippo signaling pathway, Zn^2+^ treatment promoted C2C12 cell proliferation, whereas this effect was suppressed when verteporfin was added (Supplementary Fig. S8b, c). In vivo experiments further supported these findings, showing that ZnCl_2_ accelerated skeletal muscle repair and regeneration after injury, while Zn^2+^ chelation exerted opposite effects (Supplementary Fig. S4e–h).

To further test our hypothesis that activation of somatosensory neurons contributes to Zn^2+^ release, which in turn promotes C2C12 cell proliferation through Hippo/YAP signaling; we performed additional DRG neuron–C2C12 cell coculture experiments using a microfluidic chamber system, which allows physical separation of neuronal somata and axons while enabling axon-target cell interactions^11,12^. In this setup, DRG neurons were plated in the soma compartment and infected with AAV2/9-hM3Dq. After successful infection, C2C12 cells were seeded into the axonal compartment (Supplementary Fig. S9a–c). Chemogenetic activation of DRG neurons using clozapine-N-oxide (CNO) significantly enhanced C2C12 cell proliferation and modulated the Hippo signaling activity, characterized by elevated expression of YAP, as well as reduced VGLL4 levels. Notably, this effect was abolished by the addition of the Zn^2+^-selective chelator TPEN to the axonal compartment (Supplementary Fig. S9d–f). These findings indicate that DRG neuron activation promotes C2C12 proliferation via Zn^2+^-dependent signaling. Additionally, YAP inhibitor verteporfin was also applied to the axonal compartment containing C2C12 cells. Under this condition, the chemogenetic activation of DRG neurons using CNO no longer promoted C2C12 proliferation and Hippo signal activation (Supplementary Fig. S9d–f). These results indicate that blocking Hippo/YAP signaling in the target cells effectively abolishes the proliferative effect induced by neuronal activation, further supporting that DRG neuron-derived Zn^2+^ promotes C2C12 proliferation through activation of Hippo/YAP signaling. Additionally, because Zn^2+^ is predominantly concentrated in NF200⁺ neurons in the DRG^4^, these findings support the notion that activation of NF200^+^ sensory neurons contributes to Zn^2+^ release, which in turn promotes C2C12 proliferation through Hippo/YAP signaling.

In this study, we show that the proliferation of MuSCs was accompanied by an increase in NF200^+^ LTMRs fibers during muscle regeneration. 3D imaging analysis revealed that MuSCs localize closer to these sensory nerve fibers after injury. Disruption of the afferent branch of the sciatic nerve delays MuSC proliferation and muscle regeneration, underscoring the critical role of sensory innervation. Furthermore, this study reveals that Zn^2+^, stored in NF200^+^ neurons, is released upon injury, increasing the local concentration around MuSCs and myoblasts. Mechanistically, our in vitro findings demonstrate that Zn^2+^ enhanced myoblast proliferation while inhibiting differentiation through ZIP transporters by tipping the balance towards the Hippo signaling pathway (Fig. 1n). These findings highlight a novel mechanism by which somatosensory neurons modulate myoblast proliferation and muscle regeneration. It provides promising targets for therapies of muscle degeneration diseases and age-related declines in muscle function.

## Supplementary information

Supplementary figures and methods

## Data Availability

All data presented in the main text or supplementary materials are available from the corresponding author upon reasonable request.
