# Supplementary material for: Primary somatosensory neuron-derived Zn2+ orchestrates muscle regeneration via Hippo signaling pathway
Source: Cell Discov. 2026 Jul 1;12:47. doi: 10.1038/s41421-026-00910-8 (PMC13320168; doi:10.1038/s41421-026-00910-8)
Supplement: Supplementary file 1 — Supplementary figures and methods [file 41421_2026_910_MOESM1_ESM.pdf]

Materials and Methods

Animals

The Vglut1-Cre mice (Stock No: 023527), Th-Cre mice (Stock No: 008601), Ai3 mice (Stock No: 007903), Ai27 mice (Stock No: 012567) and Ai14 mice (Stock No: 007908) were obtained from The Jackson Laboratory. Pax7-eGFP mice were generously provided by Dr. Zuoyun Wang's lab at Fudan university. Advillin-Cre mice and SNS-Cre were provided by Dr. Zhenzhong Xu's lab at Zhejiang University. C57BL/6 mice were purchased from Shanghai Lingchang Biological Technology Company. All mice were group-housed and bred in the animal facilities of Fudan University, following a 12-hour light/12-hour dark cycle at a temperature of 22 ± 1 °C. All mice used for model construction were assigned to either experimental groups or control groups. All animal procedures were conducted in accordance with the guidelines outlined in the National Institutes of Health Guide for the Care and Use of Laboratory Animals and were approved by the Animal Care and Use Committee of the Institutes of Brain Science at Fudan University.

Tissue-clearing method

Mice were intracardially perfused at 4°C with 20 ml phosphate-buffered saline (PBS) containing 10 U/ml heparin to remove blood, followed by 20 ml of 4% paraformaldehyde (PFA). Dissected tissues were post-fixed in 4% PFA at 4°C for 24 h. Samples were then processed using an organic solvent-based clearing protocol. Briefly, tissues were dehydrated through a graded methanol series (20%, 40%, 60%, 80%, and 100%, 1 h each) at 4°C, followed by an additional wash in 100% methanol. Tissues were incubated overnight at room temperature in 66 % dichloromethane (DCM)/33% methanol with shaking, washed twice with 100% methanol, and bleached overnight at 4°C in 5% hydrogen peroxide (prepared in methanol). Rehydration was carried out through a reverse methanol gradient into 1×PBS, followed by two washes in PTx.2 buffer (1 h each). For immunostaining, tissues were incubated in permeabilization solution at 37°C for 2 days, blocked at 37°C for 2 days, and subsequently incubated with primary and secondary antibodies at 37°C for 4 days each, with multiple washes in PTwH buffer between steps. After staining, tissues were dehydrated again through a

methanol series, incubated in 66% DCM/33% methanol for 3 h, washed twice with 100% DCM (15 min each, shaking), and cleared in dibenzyl ether (DBE), ensuring complete immersion to avoid oxidation. Imaging was performed using the 3D tissue clearing and imaging platform at the Department of Integrated Traditional and Western Medicine, Fudan University. Antibody compatibility with methanol pretreatment was validated prior to clearing, and image analysis was conducted using Imaris software.

Rhizotomy model

Mice were anesthetized with pentobarbital sodium (75 mg/kg i.p.), and the fur from L3 to L6 was shaved. The mice were fixed in a stereotaxic frame, and the L4-L5 region was exposed and disinfected. Using ophthalmic scissors, the peripheral branches of the DRG at the L4 and L5 levels on one side were transected. Hemostasis was achieved with a sterile cotton ball, and the muscles and skin were sutured with absorbable sutures. Four weeks later, Fluoro-Gold (Biotium,80014) was injected into the sciatic nerve to assess the success of the model construction.

Tissue processing and immunofluorescence staining

Mice were deeply anesthetized with pentobarbital sodium (75 mg/kg i.p.) and transcranial perfused with PBS, followed by 4 % paraformaldehyde in 0.01 M PBS. The TA muscle was sectioned at a thickness of 10 μm using a cryostat (Leica). The sections were incubated with primary antibodies anti-CGRP (goat, 1:1000; Bio-Rad; AB_2290729), anti-TH (rabbit, 1:1000, Millipore; AB152), anti-NF200 (mouse, 1:1000, Millipore; AB_477257), anti-$P2X_3$ (mouse, 1:1000, Millipore; AB_477257), anti-MyHC (mouse, 1:1000, Millipore; 05-716) or anti-Laminin (rat,1:1000, Abcam; Cat No. AB11576) overnight at 4°C, followed by Cy3-(1:400, Jackson ImmunoResearch Laboratories Inc. Cat No.112-165-003), Cy5-(1:400, Jackson ImmunoResearch Laboratories Inc. Cat No.715-175-151), or FITC-conjugated secondary antibodies (1:400; Jackson ImmunoResearch Laboratories Inc. Cat No.115-095-003) or DAPI (1:1000; Invitrogen; 62248). Sections were mounted and examined under Olympus confocal laser scanning microscopy.

ZnS^{AMG} staining

Mice intended for sampling were deeply anesthetized with 0.75% pentobarbital sodium at 10 μl/g body weight. After deep anesthesia, the mice were subjected to transcardiac perfusion with PBS followed by Na_2S (FD Rapid TimmStain™ Kit, Cat: Pk701), followed by perfusion with 4% paraformaldehyde (PFA) supplemented with 0.1% picric acid in PBS as separate solutions. The DRG and muscle were subsequently excised, postfixed overnight at 4°C, and subjected to dehydration through a graded series of sucrose solutions. Serial transverse sections of the muscle and DRG (14 μm) were prepared using a freezing microtome. The sections were thoroughly rinsed with 0.1 M phosphate buffer solution for 10 minutes and then immersed in an AMG incubation solution (containing 50% arabic gelatin, sodium citrate buffer, p-diphenol, and silver lactate solution) for 35 min at 32°C. To ensure consistency of zinc reactions, tissue samples from different groups were processed together in the same batch. All tissue samples were fixed, stained, and analyzed under identical conditions. Observers maintained blinding throughout the entire experimental and analysis processes to ensure the objectivity and consistency of data interpretation. Following dehydration and clearing procedures, the sections were sealed with neutral gum. Subsequently, brightfield microscopy images from ZnS^{AMG} staining section series were acquired using a Nikon microscope through a 20 × objective. The white balance was adjusted prior to image capture.

Cell culture

C2C12 cells were maintained in high-glucose DMEM supplemented with 10% fetal bovine serum (FBS), 1% penicillin-streptomycin (P/S), 4.5 g/L D-glucose, L-glutamine, and 110 mg/L sodium pyruvate, at 37°C in a humidified incubator with 5% CO_2. Human MuSCs were isolated from human biopsies by FACS sorting $CD29^+$ $CD56^+$ cells. Human MuSCs were cultured in DMEM/F12 medium containing 5ng FGF and 15% FBS, 1% penicillin-streptomycin (P/S) at 37°C in a humidified incubator with 5% CO_2. To induce differentiation, the growth medium was replaced with differentiation medium containing 2% horse serum in place of FBS.

Intrathecal injection of ZX1

ZX1 (Cat. No: 07-0350) was purchased from Strem Chemicals. ZX1 is a Zn^{2+} chelator that selectively binds free Zn^{2+}. For intrathecal injection, ZX1 (100 μM) was delivered (10 μl) via a spinal cord puncture using a 30-gauge needle between the L5 and L6 levels.

Intramuscular injection of ZX1

For local chelation of Zn^{2+}, 50 μL of ZX1 (100 μM) was injected into the tibialis anterior (TA) muscle using an insulin syringe at the same site within 15–30 min following CTX injection.

CTX injury

Muscle injury was induced by the injection of CTX (Sigma-Aldrich, 217503) to TA muscles. A total of 50 μl of CTX was administered per mouse using an insulin syringe, with equal distribution across the upper, middle, and lower regions of the TA muscle. 6-8-week-old mice were anesthetized with 0.75% sodium pentobarbital, and the TA region was sterilized with 75% ethanol. Excess hair was carefully removed. Successful injection was confirmed by the appearance of a localized swelling. TA muscles were collected and analyzed at 5, 7, and 21 days after CTX injury.

RT-PCR

Cells were harvested and RNA was extracted using Trizol reagent. The quantity and purity of the total RNA were assessed using a NanoDrop ND-1000 spectrophotometer (NanoDrop Technologies Inc.). Reverse transcription of RNA (0.5-2 μg) was performed using the 5 × All in One RT MasterMix (abm, G492). PCR amplification was carried out by adding primers, and the products were analyzed by gel electrophoresis.

Genotyping

To purify genomic DNA, mouse ear tissue was collected and digested in a solution containing 25 mM NaOH and 0.2 mM EDTA at 95 °C for 1 hour, followed by neutralization with 40 mM Tris-HCl (pH 5.5). Genotyping of Pax7-eGFP mice was performed using the primer pair 5’-acgtaaacggccacaagttc-3’ and 5’-gtcctccttgaagtcgatgc-3’, resulting in a mutant band size of 388 bp. The primers for genotyping VGluT1-cre mice were 5’-ccctaggaatgctcgtcaag-3’, 5’-atgagcgaggagaagtgtgg-3’ and 5’-gtggaagtcctggaaactgc-3’ with band size 344 bp. The

primers for genotyping TH-cre mice were 5'-gcggtctggcagtaaaaactatc-3', 5'-gtgaaacagcattgctgtcactt-3' with band size 100 bp.

Single-cell RNA sequencing data analysis

The single-cell RNA sequencing data analyzed in this study were obtained from a publicly available dataset accessible through GEO (accession number GSE143435). Quality control was performed to exclude cells with low gene detection (< 200 detected genes), cells with high mitochondrial gene content (> 5%), and genes expressed in fewer than 3 cells. The filtered dataset was then normalized and log-transformed using Seurat v5.0, and downstream analyses were conducted with Seurat and other standard bioinformatics tools.

Cell viability assay

A 96-well plate was prepared by adding 100 μl of complete culture medium to the blank wells (Ab), 100 μl of cell suspension containing approximately 2000 C2C12 cells in the control wells (Ac), and 100 μl of cell suspension with varying concentrations of the drug (each containing approximately 2000 C2C12 cells) in the experimental wells (As). Each condition was tested in triplicate. The plate was incubated for 12 hours at 37°C in a 5% CO2 incubator. After incubation, 10 μl of CCK-8 reagent was added to each well, and the plate was incubated for an additional 2 hours. Absorbance (OD) values were measured using a microplate reader. Cell viability was calculated using the following formula: Cell viability = [(As - Ab) / (Ac - Ab)] × 100%.

Fiber size distribution and fusion index

Cross-sectional area of the myofibers was calculated on section images obtained from TA muscles using Image pro plus. To quantify the fusion of C2C12 cells, total cell nuclei and nuclei within myotubes were counted using Image Pro Plus. Fusion index was calculated as the number of nuclei in myotubes divided by the total number of nuclei counted.

Immunoblotting analysis

Total proteins were performed using lysis buffer (62.5mM Tris-HCL, PH=6.8, 2% SDS,10% Glycerine, 5% β-Mercaptoethanol, 0.05% bromophenol blue, 1% protease inhibitor cocktail). The proteins were separated by electrophoresis and transferred to

0.45μm PVDF membranes. Membranes were blocked in 5% milk and then incubated with the primary antibodies. The primary antibodies used anti-Ki67 (1:1000, Abcam, ab16667), human anti-Pax7 (1:1000, DSHB, Pax7), mouse anti-MyHC (1:1000, upstate, 05-716), mouse anti-MyoG (1:1000, Santa Cruz, sc-12732), mouse anti-MyoD (1:1000, Santa Cruz, sc-32758), rabbit anti-VGLL4 (1:1000, ABclonal, A18248), rabbit anti-VGLL4 (1:1000,lifespan, LS-C497286), rabbit anti-YAP (1:1000, CST, 14074S), rabbit anti-p-YAP (1:1000, CST, 4911S), and anti-GAPDH (1:5000 ABclonal #AC002).

Quantification and statistical analysis

All data are presented as bar graphs indicating mean ± SEM (standard error of the mean). Dots in bar graphs and boxplots represent individual values per mouse or per image, horizontal line indicates average. GraphPad Prism scientific software version 8.4.0 was used for statistical analysis. For normally distributed, related samples with equal variances, unpaired t test and paired t test was used to compare two groups. Conversely, samples with unequal variances via Welch's t-test. Statistical significance was determined using both nonparametric (Mann–Whitney, Kruskal–Wallis test with Dunn's multiple-comparisons post hoc test) and parametric methods (One-way ANOVA followed by Dunnett's multiple comparisons, Two-way ANOVA with Sidak's multiple comparisons Bonferroni post hoc analysis) due to differences in the normality and sample size of the data. The specific tests used to analyze each data set is indicated within the individual figure legends, with results expressed as mean ± SEM Significance levels are indicated as *$p < 0.05$, **$p < 0.01$, ***$p < 0.001$.

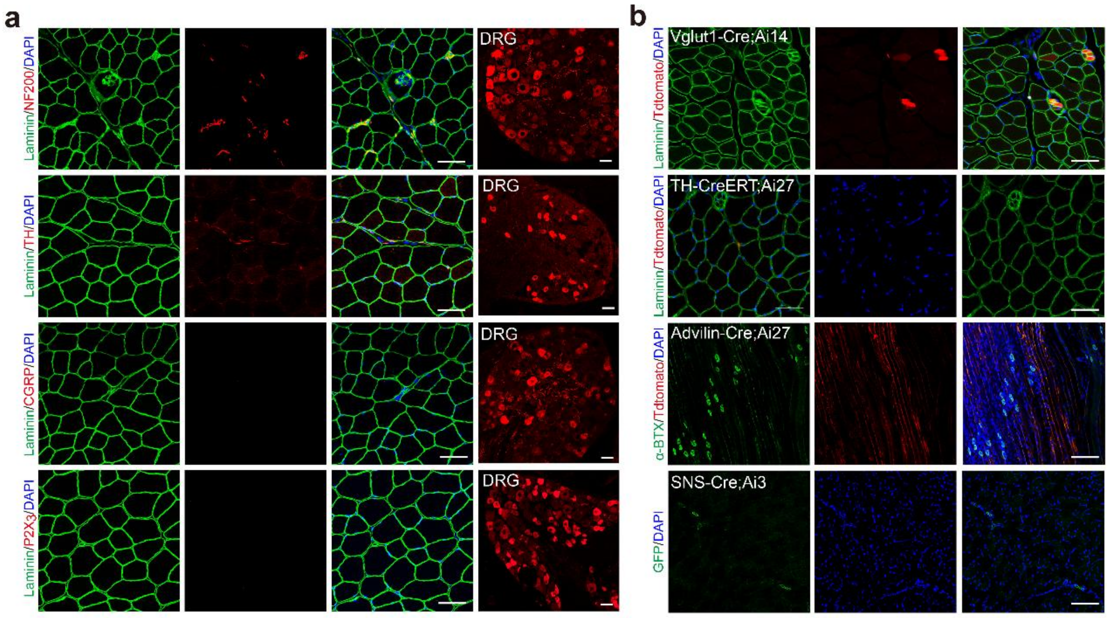

Supplementary Fig. S1 Sensory nerves innervate in TA. a. Representative IF images of the sensory nerves of the TA muscle and dorsal root ganglia (DRG) of mice. Laminin (green) double labelled with different neuronal markers CGRP, $P2X_3$, TH or NF200 (red), and DAPI (blue) represents the nucleus. DRG sections are included as positive controls for each neuronal marker. Scale bars = 50 μm. **b.** Representative IF images of the TA muscle in Vglut1-Cre; Ai14 mice; TH-CreERT; Ai27 mice; Advilin-Cre; Ai27 mice; and SNS-Cre; Ai3 mice. Scale bars = 50 μm.

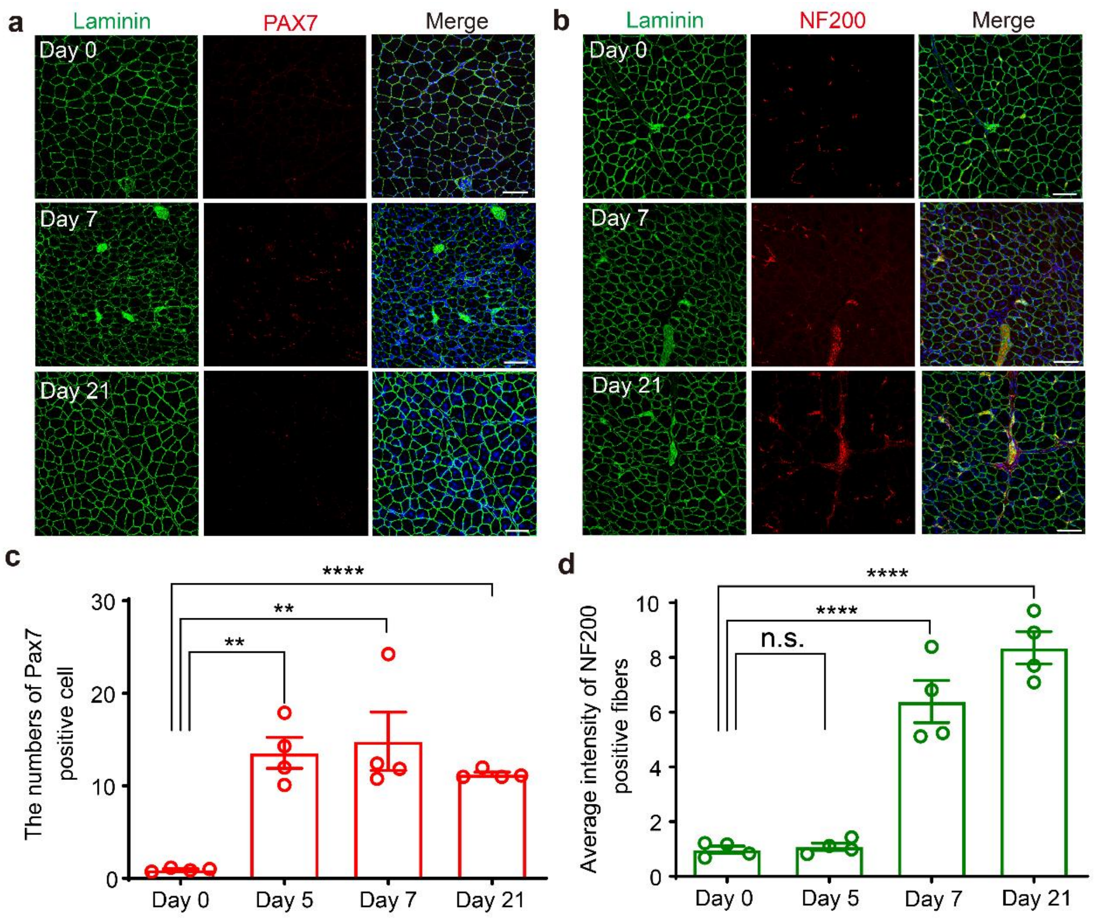

Supplementary Fig. S2 MuSCs change during muscle injury for repair and NF200^{+} neurons increase after muscle injury. a. Representative IF images of MuSCs after different days of CTX injury in mice. Scale bars are 50 μm. **b.** Representative IF images of NF200 after different days of CTX injury in mice. Scale bars = 100 μm. **c.** The number of Pax7 positive cells after different days of CTX injury in mice. Data are shown as mean ± SEM, n = 4 mice. **d.** The number of NF200 positive cells after different days of CTX injury in mice. Data are shown as mean ± SEM, n = 4 mice.

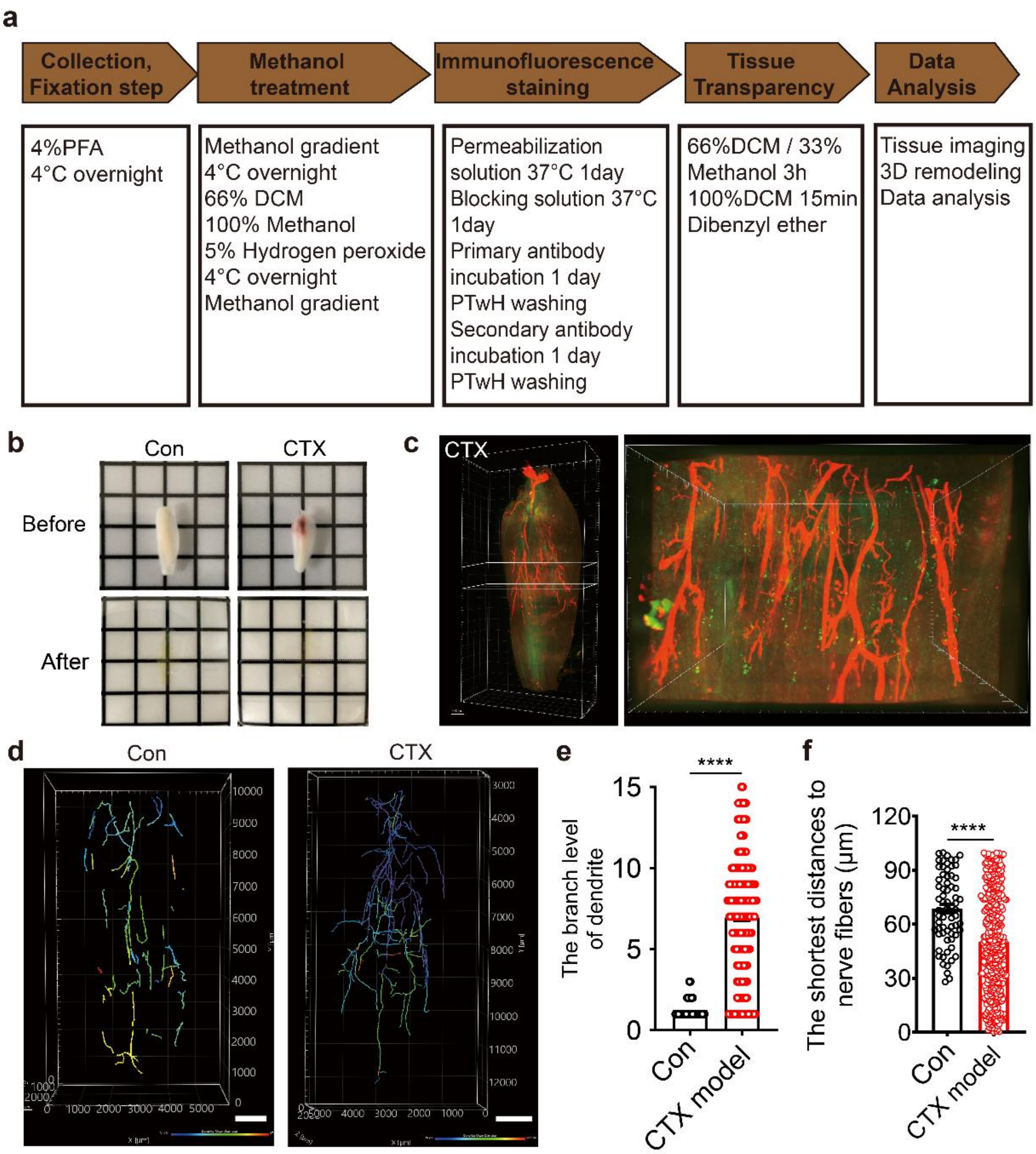

Supplementary Fig. S3 Organizational transparency and 3D modeling reveal that sensory neuron innervation increases after muscle injury. a. Experimental flowchart for organizational transparency. **b.** Schematic diagram of TA muscle after tissue clearing. **c.** 3D images of TA muscles on both sides of Pax7-EGFP mice after tissue clearing. The right side of C is a partial enlarged view of the dotted line on the left. Scale bars = 1000 μm. **d.** Schematic diagram of 3D modeling of sensory nerve. Scale bars = 1000 μm. **e.** Quantification of the level sensory branches following muscle injury (****p<0.0001, Unpaired t-test). **f.** Quantification of the shortest distance between MuSCs and nerve fibers after muscle injury (****p<0.0001, Unpaired t-test).

Supplementary Figure 4 related to Main Figure (c-f)

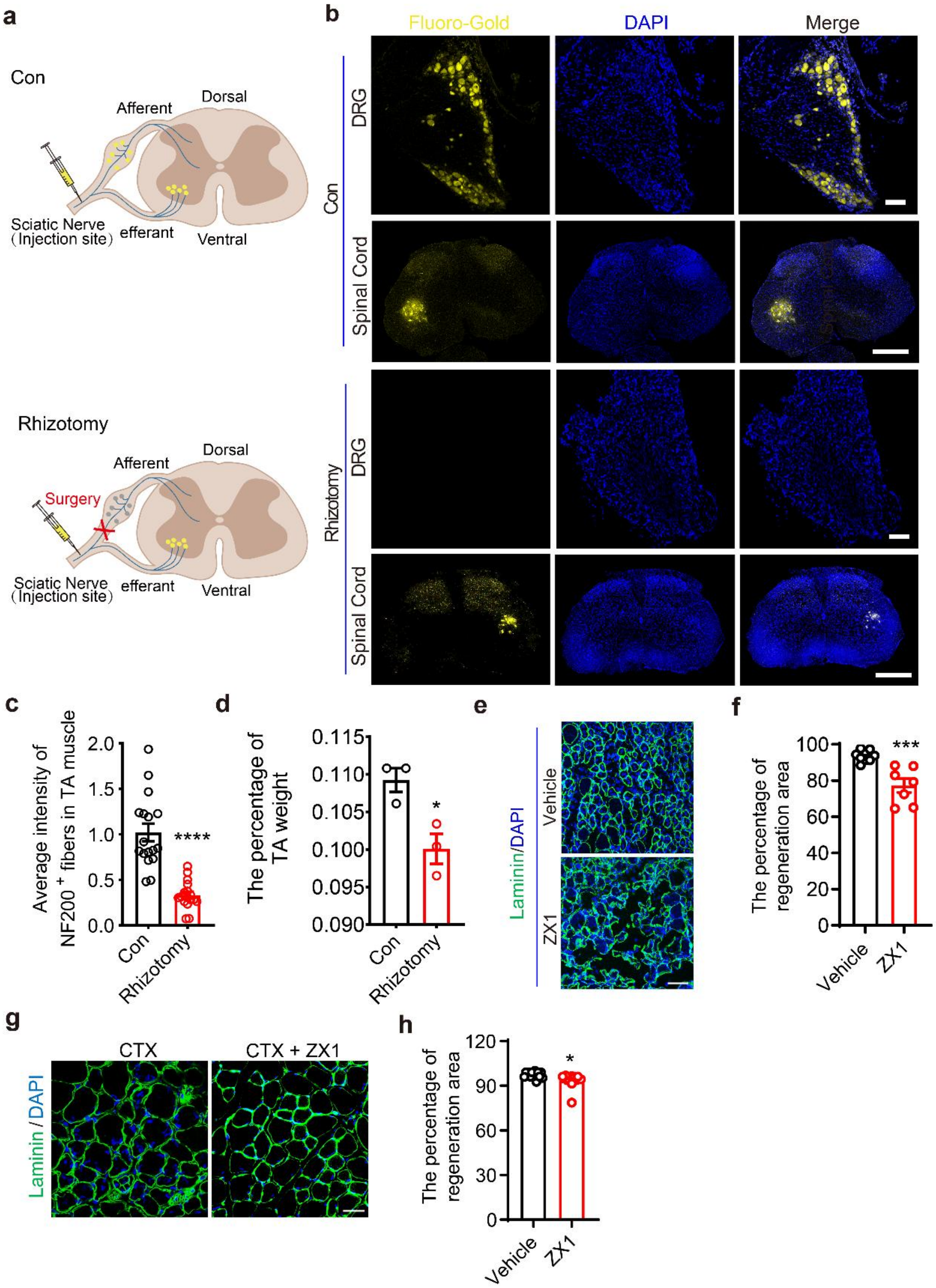

Supplementary Fig. S4 Construction of the Rhizotomy model confirm the innervation of sensory nerves. a. Schematic illustration of the surgical procedure **b.** Representative immunofluorescence (IF) images of the spinal cord and DRG after rhizotomy. Scale bars are 500 μm in spinal cord and 100 μm in DRG. **c.** The average

intensity of $NF200^+$ nerve fibers in TA after rhizotomy (*****p*<0.0001, Unpaired *t*-test). **d.** The percentage of TA weight decreased after surgery (**p*<0.05, Unpaired *t*-test). **e.** Cross-sectional comparison of tibialis anterior (TA) muscle at 5 days post-CTX injury following intrathecal injection of vehicle or ZX1. Scale bars = 100 μm. **f.** Quantification of TA muscle regeneration area at 5 days post-CTX injury following ZX1 treatment compared to vehicle (****p*<0.001, Unpaired *t*-test). **g.** The representative IF images of tibialis anterior (TA) muscle at 5 days post-CTX injury following injection of vehicle or ZX1. Scale bars = 20 μm. n = 4 mice. ZX1 concentration = 50 μM. **h.** Cross-sectional comparison of tibialis anterior (TA) muscle at 5 days post-CTX injury following intrathecal injection of vehicle or ZX1. ZX1 concentration = 50 μM. (**p*<0.05, Unpaired *t*-test).

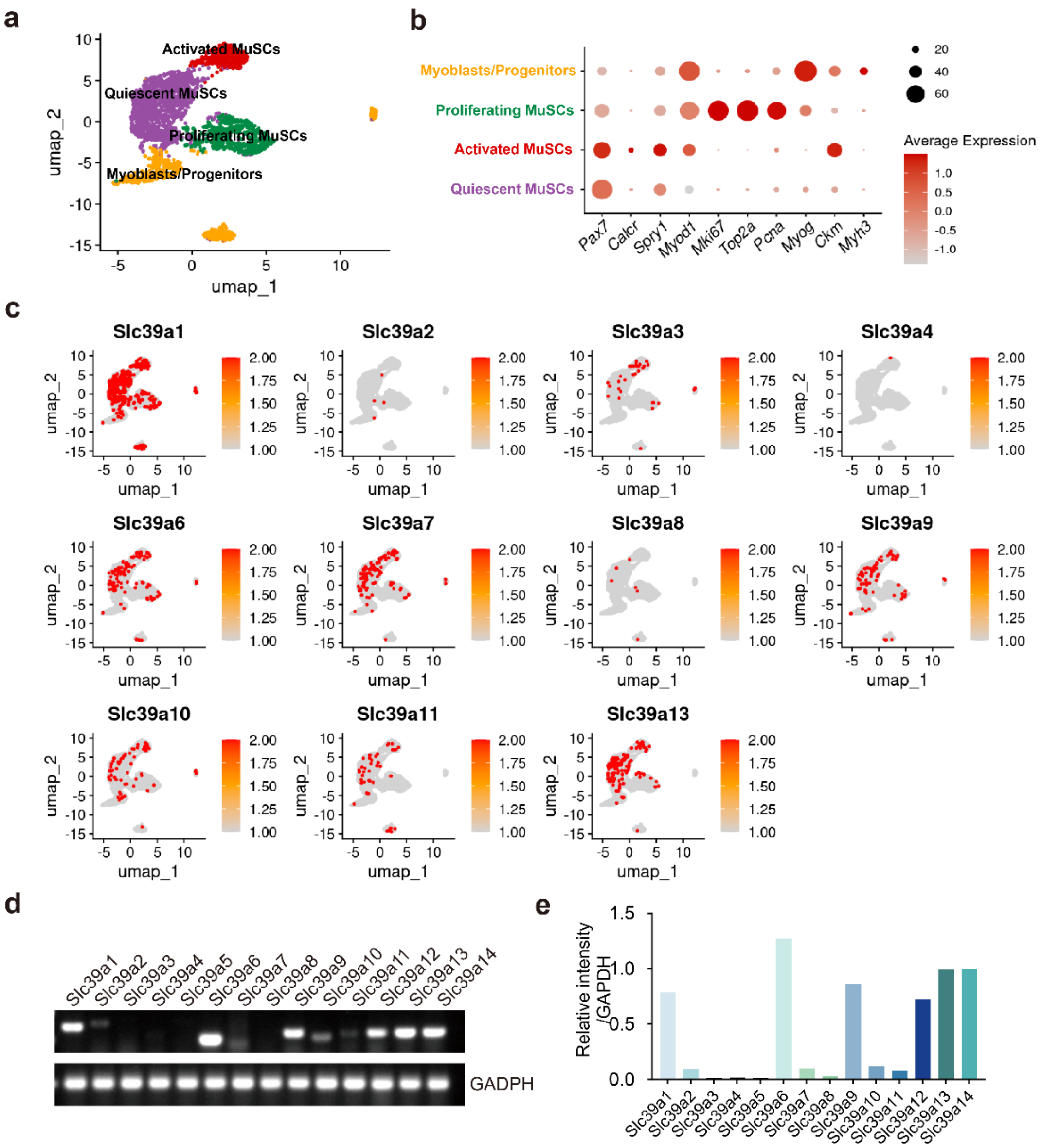

Supplementary Fig. S5 Zinc ion transporters show high expression in MuSCs. a. UMAP visualization of quiescent, activated, proliferating MuSCs and myoblasts/progenitors. **b.** Dot plot showing marker gene expression used for MuSC cluster annotation. **c.** Expression of zinc ion transporter Zip family in muscle stem cells. **d.** RT-PCR detection of zinc ion transporters in C2C12 cells. **e.** RT-PCR detection of zinc ion transporters in C2C12 cells.

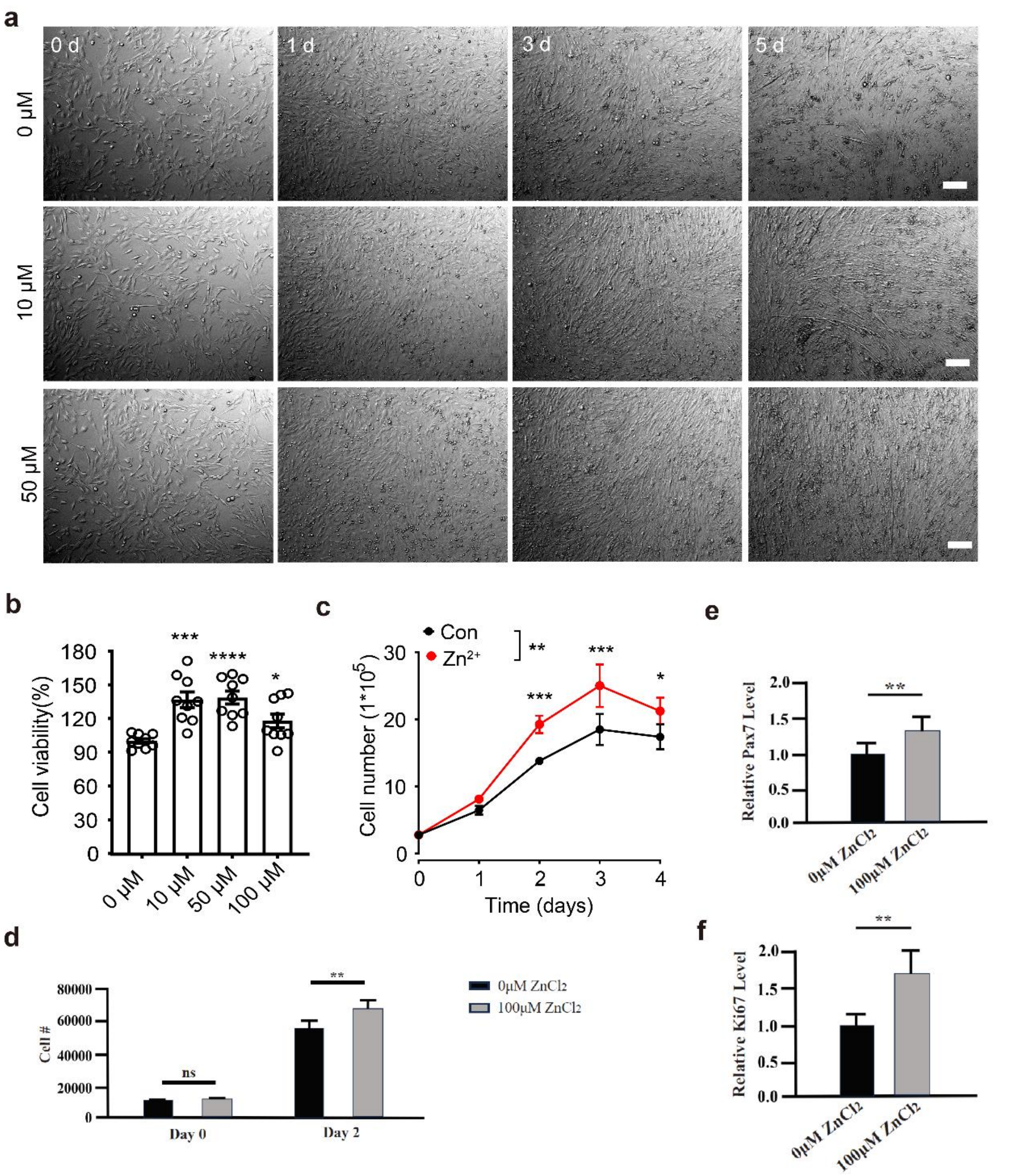

Supplementary Fig. S6 Zn^{2+} promotes the differentiation of C2C12 cells. a. The differentiation state of C2C12 cells in different culture time under the action of different concentrations of Zn^{2+}. Scale bars = 200 μm. **b.** Cell viability of C2C12 cells under the action of different concentrations of Zn^{2+} (*p<0.05, ***p<0.001, ****p<0.0001, One-way ANOVA) **c.** The number of C2C12 cells increased under the action of Zn^{2+}, Zn^{2+} concentration = 50 μM (*p<0.05, **p<0.01, ***p<0.001, Two-way ANOVA) **d.** Statistical analysis of cell numbers after 100μM $ZnCl_2$ treatment (n=3, **p<0.01, unpaired *t*-test) **e-f.** RT-qPCR of the expression level of Pax7 and Ki67 after 48 h Zn^{2+} treatment of human MuSCs (n=3, **p<0.01, Unpaired *t*-test).

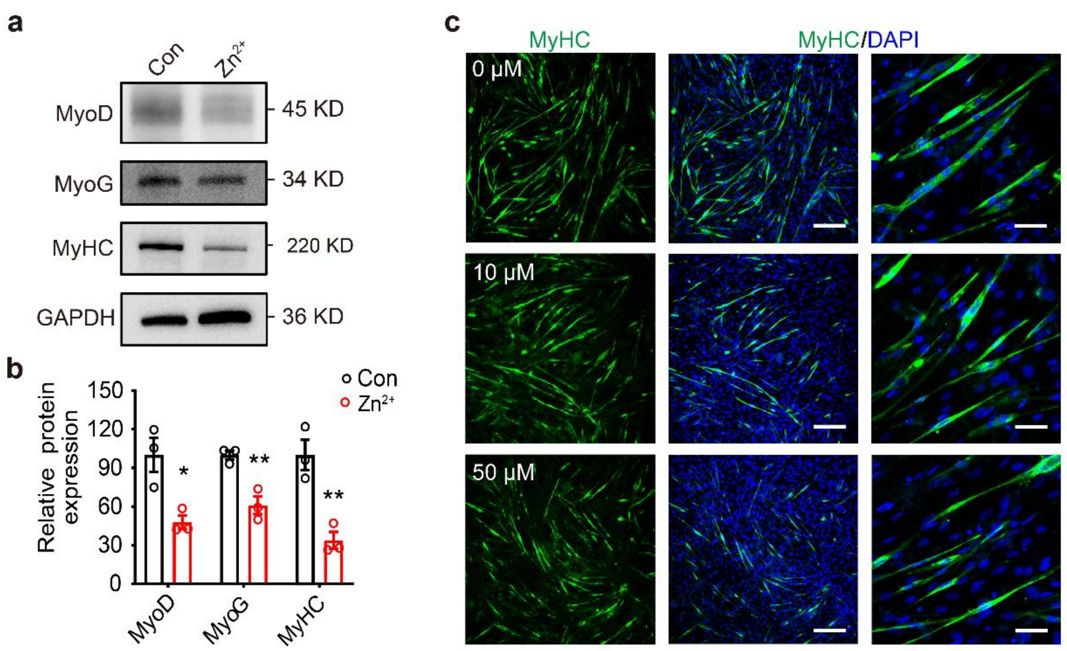

Supplementary Fig. S7 Zn^{2+} inhibits the proliferation of C2C12 cells. a. Under the action of Zn^{2+}, the protein change of MyoD, MyoG and MyHC in the proliferation stage of C2C12 cells. **b.** Statistical analysis of sequence marker proteins in the differentiation stage of C2C12 cells under the action of Zn^{2+} (*p<0.05, **p<0.01, Unpaired *t*-test). **c.** The representative IF images of MyHC in C2C12 cells under the action of different concentrations of zinc ions. The scale bars are 200 μm in the first two columns and 100 μm in the third column.

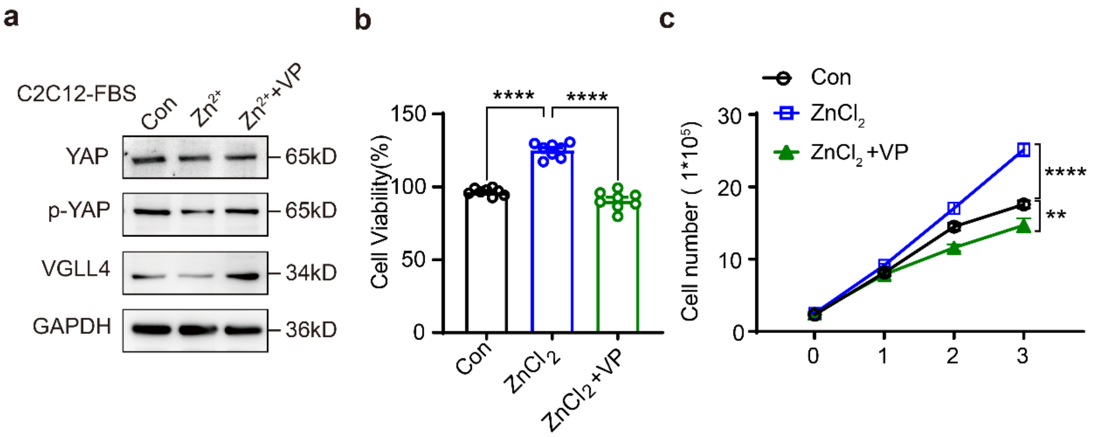

Supplementary Fig. S8 YAP inhibitor verteporfin blocked the effect of Zn^{2+} on C2C12 cells. a. Changes in Hippo pathway core protein YAP, p-YAP, and VGLL4 expression during proliferation stage under Zn^{2+} or Zn^{2+} in combination with verteporfin treatment. Vehicle, Zn^{2+} or Zn^{2+} in combination with verteporfin treatment. **b.** Cell viability of C2C12 cells treated with Zn^{2+} and in combination with verteporfin. (**p*<0.05, ****p*<0.001, *****p*<0.0001, One-way ANOVA.) **c.** The number of C2C12 cells increased under the action of Zn^{2+} and in combination with verteporfin, Zn^{2+} concentration = 50 μM; verteporfin concentration = 50 μg/ml (**p*<0.05, ***p*<0.01, ****p*<0.001, *****p*<0.0001, Two-way ANOVA.)

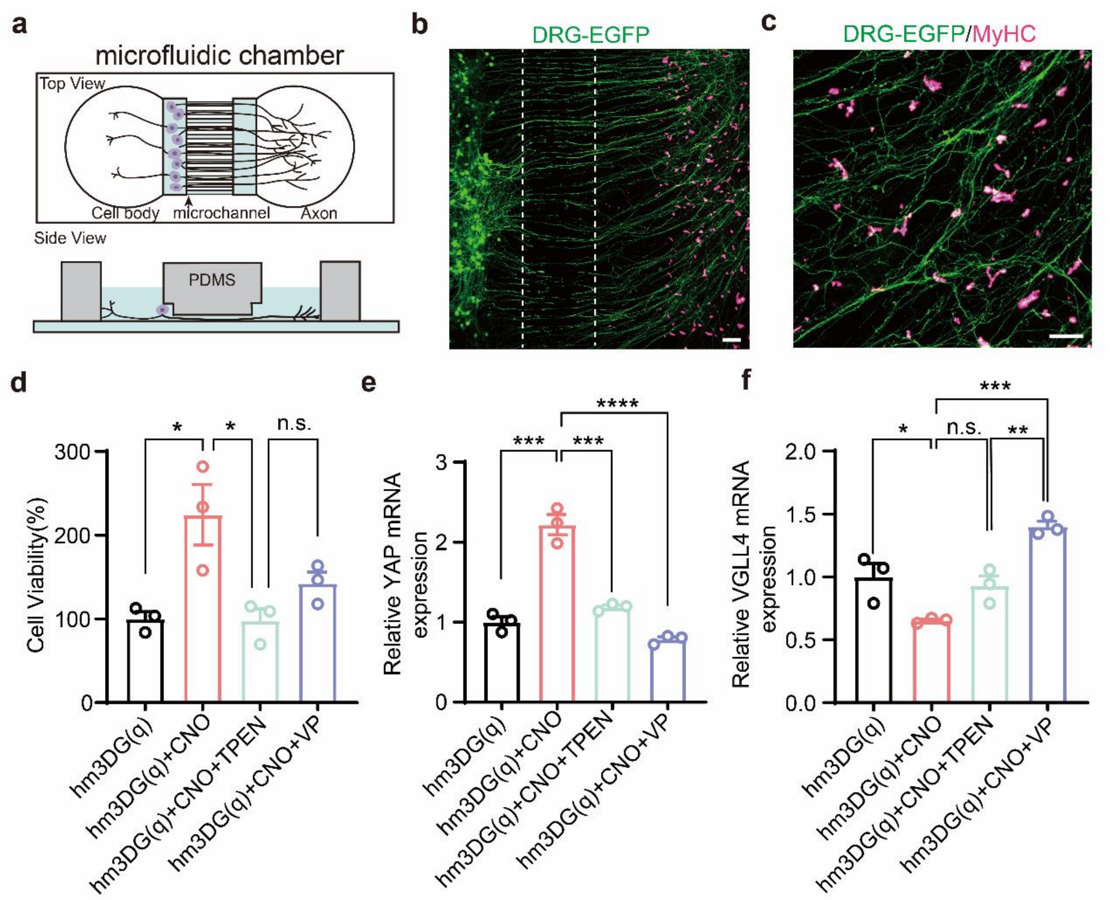

Supplementary Fig. S9 Sensory neuron-released Zn^{2+} affects the Hippo pathway status in co-cultured C2C12 cells. a. Schematic diagram for microfluidic chamber. **b-c.** Representative images of coculture of C2C12 cells ($MyHc^{+}$) with DRG axons. d. Cell viability measured by CCK-8 assay. **e-g**. Changes in the relative mRNA expression levels of the Hippo pathway members YAP and VGLL4. Verteporfin concentration = 50 μg/ml (*p<0.05, **p<0.01, ***p<0.001, ****p<0.0001, Two-way ANOVA.)
